# Association between peroxisome proliferator-activated receptor-alpha, delta, and gamma polymorphisms and risk of coronary heart disease: A case–control study and meta-analysis: Erratum

**DOI:** 10.1097/MD.0000000000005949

**Published:** 2017-01-13

In the article “Association between peroxisome proliferator-activated receptor-alpha, delta, and gamma polymorphisms and risk of coronary heart disease: A case–control study and meta-analysis”,^[1]^ which appeared in Volume 95, Issue 32 of *Medicine*, the following acknowledgment was originally omitted:

This work was supported by a grant from the National Natural Science Foundation of China (No. 81302455).
